# Level of muscle regeneration in limb-girdle muscular dystrophy type 2I relates to genotype and clinical severity

**DOI:** 10.1186/2044-5040-1-31

**Published:** 2011-10-05

**Authors:** Thomas O Krag, Simon Hauerslev, Marie Louise Sveen, Malene Schwartz, John Vissing

**Affiliations:** 1 Neuromuscular Research Unit, Department of Neurology Rigshospitalet, University of Copenhagen, Denmark; 2 Department of Clinical Genetics, Rigshospitalet, University of Copenhagen, Denmark

## Abstract

**Background:**

The balance between muscle regeneration and ongoing degeneration is a relationship that greatly influences the progression of muscular dystrophy. Numerous factors may influence the muscle regeneration, but more information about the relationship between genotype, clinical severity and the ability to regenerate is needed.

**Methods:**

Muscle biopsies were obtained from the tibialis anterior muscle, and frozen sections were stained for general histopathological and immunohistological evaluation. Differences between groups were considered statistical significant at *P *< 0.05 using Student's unpaired *t*-test.

**Results:**

We found that all patients with limb-girdle muscular dystrophy type 2I (LGMD2I) had a large number of internally nucleated fibers, a sign of previous regeneration. The level of expression of muscle-specific developmental proteins, such as neonatal myosin heavy chain (nMHC) and myogenin, was related to the clinical severity. Additionally, we found that the majority of nMHC-positive fibers did not stain positively for utrophin in patients who were compound heterozygous for the L276I mutation, suggesting that the predominant form of regeneration in these patients is fiber repair rather than formation of new fibers. Double staining showed that many smaller nMHC-positive fibers were positive for antibodies against the glycosylation on α-dystroglycan, suggesting that such glycosylation may be a result of muscle regeneration.

**Conclusion:**

Severely affected patients with LGMD2I have a high level of muscle degeneration, which leads to a high rate of regeneration, but this is insufficient to change the imbalance between degeneration and regeneration, ultimately leading to progressive muscle wasting. Detailed information regarding the level and rate of muscle regeneration and potential obstructions of the regenerative pathway should be of use for future therapies involving satellite-cell activation.

## Background

Limb-girdle muscular dystrophy type 2I (LGMD2I) was molecularly characterized in 2001 as caused by mutations in the gene for fukutin-related protein (FKRP) [1]. It has been suggested that FKRP is involved in glycosylation of α-dystroglycan (α-DG) [2], a protein that stabilizes the link between the contractile apparatus and the extracellular matrix (ECM) [3]. Although it is believed to possess enzymatic properties, the exact function of FKRP has not yet been elucidated. It has been suggested that mutations affecting the translocation of FKRP from the endoplasmic reticulum to the Golgi apparatus invariably lead to severe phenotypes, whereas mutations that do not affect lodging of FKRP in the Golgi membrane may lead to moderate phenotypes [4]. However, other investigators have suggested that the main pathogenic mechanism relates to a lack of interaction between FKRP and its substrate(s) in the Golgi [5].

In Denmark, LGMD2I accounts for 40% of the total number of recessively inherited cases of LGMD2 [6]. Most patients with LGMD2I are homozygous for the mutation L276I (826A>C transversion). This genotype is generally associated with a mild phenotype, whereas patients who are compound heterozygous for this mutation generally display a more severe phenotype [7,8].

There is no known substitute for FKRP, and loss of its function in LGMD2I leads to degeneration of myofibers and secondary loss of proteins that are important for structure and function, such as α-DG and merosin [9,10]. To counterbalance degeneration, a muscle must regenerate its fibers, and establishing the regenerative response to muscle damage in muscular dystrophies may provide important clues to therapeutic interventions.

In general, muscle damage leads to activation of quiescent satellite cells residing between the sarcolemma and basal lamina. Once the satellite cell has fused with the myofiber, its nucleus migrates to the site that requires repair. This migration is visible histologically as internally nucleated fibers, and counting these has traditionally been one way of determining muscle regeneration [11]. However, internally nucleated fibers may not fully explain the temporal pattern of a regenerative response to ongoing degeneration, because internal nuclei can reside in the cytoplasm of myofibers for several months.

In this study, we determined the temporal regenerative response to muscle degeneration in patients with LGMD2I, using developmental and myogenic markers that are only expressed for days or weeks, such as MyoD (0 to 2 days) and myogenin (1 to 7 days), neonatal myosin heavy chain (nMHC, 1 to 3 weeks) and utrophin (weeks to months) [12-14]. We assessed the recent regeneration in muscle fibers of patients homozygous and compound heterozygous for the L276I mutation, and correlated this with the age at biopsy and duration of LGMD2I in patients, as the capacity to regenerate normally decreases with age [15].

## Methods

The study was approved by the Scientific Ethics Committee of Copenhagen and Frederiksberg Counties approved this study, and all patients provided informed consent.

### Patients

In total, 22 patients with LGMD2I participated in the study (Table 1). Clinical onset was defined as the first time patients noticed symptoms such as decreased walking distance, muscle pain after physical exertion, trouble walking up stairs, rising from a seated or crouched position, and tendency to fall. Of the 22 patients with LGMD2I, 17 (eight male, nine female; mean ± SD age 44 ± 14 years, range 23 to 65 years) were homozygous for the L276I mutation and had disease onset at 19 ± 11 years of age (range 2 to 36 years). Six of these patients had disease onset in childhood, and the rest had a later onset. Duration of illness was defined as the period from age at onset to age when the biopsy was taken. The remaining five patients (one male, four female; mean ± SD age 27 ± 12 years, range 13 to 40 years,) were heterozygous for the L276I mutation, and their disease onset occurred at 6 ± 4 years of age (range 3 to 12 years). Muscle morphology was evaluated in samples from the tibialis anterior. The strength of ankle dorsal flexion was therefore tested by dynamometry and used as a measure of clinical severity for the biopsied muscle [16]; it was normalized to the ankle dorsal flexion (196 ± 52 N) determined in healthy subjects (14 male, 13 female; age 32 ± 12 years, range 16 to 59 years). From this group, six healthy controls (three female, three male; age 45 ± 11 years, range 31 to 55 years), formed the control group for all histology and immunohistology in the study.

**Table 1:** Clinical and genetic presentation of patients with limb-girdle muscular dystrophy type 2I

Mutation	Gender, M:F	Age at biopsy, years	**Onset of disease**^**a**^**, years**	Lost ambulation	Respiratory aid
L276I/L276I	9:8	44 ± 14	19 ± 11	3 patients	No

L276I/Y307N	M	35	7	Yes	BiPAP^b^

L276I/L202Q	F	40	4	Yes	Respirator

L276I/A157P	F	16	3	No	No

L276I/P462S	F	31	12	Yes	No

L276I/Y307N	F	13	3	Yes	BiPAP

^a^Age when patient first reported symptoms.

^a^Bilevel positive air pressure.

### Muscle biopsy

Muscle biopsies were obtained from the tibialis anterior muscle by percutaneous needle biopsy technique using a 5-mm Bergstrøm needle. Biopsies were snap-frozen in isopentane, cooled in liquid nitrogen, and stored at -80°C before being cut into sections on a cryostat.

### Histology and immunohistochemistry

Sections 10 to 12 μm thick were stained with hematoxylin and eosin (H&E) for general histopathological evaluation and assessment of internal nuclei, defined as non-peripheral internal nuclei. Full sections were evaluated for the percentage of internal nuclei, necrotic and ring fibers, and the level of fibrosis and muscle-fat infiltration. A myofiber was considered internally nucleated if at least one nucleus was non-peripheral. A three-point visual analog score was used to assess pathological severity as described previously by Fanin *et al*. (1 = active dystrophic process (marked increase in fiber-size variability, active degeneration and regeneration, marked increase in connective tissue); 2 = moderate dystrophic process (marked increase in fiber-size variability, increased internal nuclei, few degenerating and regenerating fibers, slight increase in connective tissue); and 3 = mild myopathic picture (moderate increase in fiber-size variability, increase in internal nuclei)) [17]. Two investigators (SH and TOK), who were blinded to genotype, assessed all biopsies.

For immunohistochemistry, all sections were fixed in a 1:1 mixture of acetone and methanol or in 4% paraformaldehyde (for MyoD), and were subsequently blocked using 3% FCS in PBS, then stained. Primary antibodies were diluted in blocking buffer (1:100). To assess the number of myofibers presently undergoing regeneration, sections were double-stained with antibodies to nMHC (Vector Laboratories, Burlingame, CA, USA) and utrophin (Santa Cruz Biotechnology, Santa Cruz, CA, USA). Activated satellite cells were visualized with MyoD antibodies (Vector Laboratories), and differentiated satellite cells (myoblasts) were visualized with myogenin antibodies (clone F5D, developed by Dr Woodring E. Wright, Developmental Studies Hybridoma Bank, under the auspices of the NICHD and maintained by The University of Iowa, Iowa City, IA, USA). Size and shape was used as criteria to separate myogenin-positive atrophic fibers from myogenin-positive regenerating fibers. All positive fibers were counted, and calculated as a percentage of the total number of fibers. To determine the effect of loss of function of FKRP, we assessed the presence of glycosylated α-DG (clone VIA4-1, Upstate, Charlottesville, VA, USA), following the manufacturer's protocol. Alexa 488 and Alexa 594 (Invitrogen, Carlsbad, CA, USA) secondary anti-mouse and anti-goat antibodies were used at1:500 dilution in blocking buffer. A microscope with epifluorescence was used for all microscopy (80i; Nikon, Tokyo, Japan), and pictures were taken using a 10× objective. Pictures were merged for quantification of the entire section.

### Statistical analysis

Statistical significance between groups was determined using Student's unpaired *t*-test and *P *< 0.05 was considered significant. All numbers provided are mean ± SD. Linear regression analysis was performed for internal nuclei versus age (seventeen L276I homozygous patients and five heterozygous patients) and non-linear regression analysis for nMHC versus age and duration of illness (twelve L276I homozygous patients and five compound heterozygous patients; biopsies from five homozygous patients were not available for immunohistochemical staining, as only H&E-stained sections were available). *P*-values were determined from a Pearson correlation coefficient table of critical values.

## Results

### Morphology

**Figure 1:**
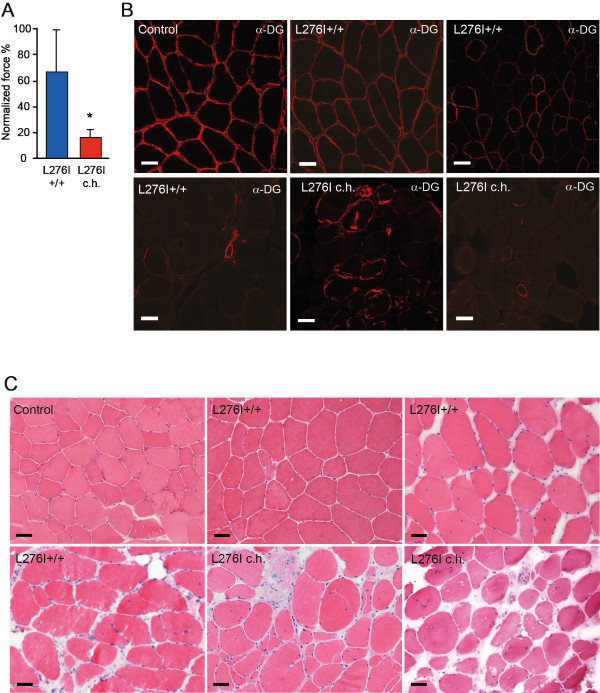
**Differences in muscle strength and morphology between patients homozygous and those compound heterozygous for the L276I mutation**. (A) Normalized ankle dorsal flexor force was reduced in L276I compound heterozygous (L276I c.h.) versus homozygous (L276I+/+) patients (*P < 0.003). (B) Immunostaining for the fukutin-related protein (FKRP) substrate, α-dystroglycan (α-DG), showed a uniform presence in the sarcolemma of healthy controls, whereas it varied in homozygous patients, reflecting the heterogeneity of the disease, and was mostly restricted to newly formed fibers and ring fibers in compound heterozygous patients. (C) Compared with normal muscle (control), muscle of both L276 homozygous and compound heterozygous patients displayed internal nuclei, fibrosis and small newly formed regenerating fibers. The heterogeneous nature of the disease was evident for the patients homozygous for the L276I mutation. (B, C) Bars = 50 μm.

**Figure 2:**
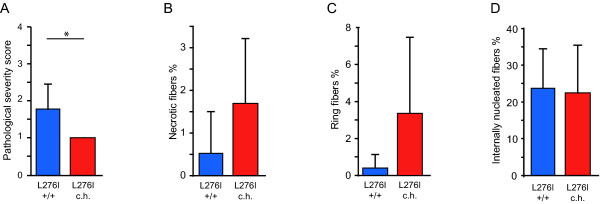
**Histopathological evaluation of patients with LGMD2I**. (A) The pathological severity score (1 = extensive degeneration of the muscle, 2 = moderate pathological changes in morphology, 3 = mild morphological changes), reflecting the overall morphology of the muscle in terms of degeneration, regeneration, and extent of fatty fibrous-tissue infiltration, shows that there was a significant difference between the group of patients with LGMD2I homozygous (blue bar) and the group compound heterozygous (red bar) for the L276I mutation. (B-D) There was no significant difference in number of (B) necrotic fibers, (C) ring fibers or (D) internally nucleated fibers between patients homozygous (blue bar) and those compound heterozygous (red bar) for the L276I mutation. *P < 0.05.

The muscle-biopsy sections from the 22 patients had an average of 729 ± 409 myofibers, whereas those from the six healthy subjects had 1351 ± 400. We found a four-fold lower (*P *< 0.00002) normalized ankle dorsal flexor force for patients who were compound heterozygous compared with those homozygous for the L276I mutation (Figure 1A). Patients homozygous for the L276I mutation had a rather varied pattern of glycosylated α-dystroglycan staining in the sarcolemma, ranging from most fibers being positive, to an interspersed pattern with half the fibers positive and half negative, to very few positive fibers (Figure 1B). In patients who were compound heterozygous for the L276I mutation, glycosylated α-dystroglycan was seen in less than 20% of fibers, with ring fibers staining strongly (Figure 1B). Compared with healthy normal muscle, H&E-stained muscle sections from homozygous patients revealed a mild to severe pathology, whereas the muscle morphology of compound heterozygous patients invariably showed severe pathology (Figure 1C). Pathological severity was significantly different between groups (Figure 2A), but there were no significant differences in numbers of necrotic fibers, ring fibers or internally nucleated fibers between the homozygous and compound heterozygous patients (Figure 2B-D). Fiber splitting was seen in three of the homozygous patients and in all of the compound heterozygous patients. Healthy subjects did not display any abnormal morphology.

### Regeneration

**Figure 3:**
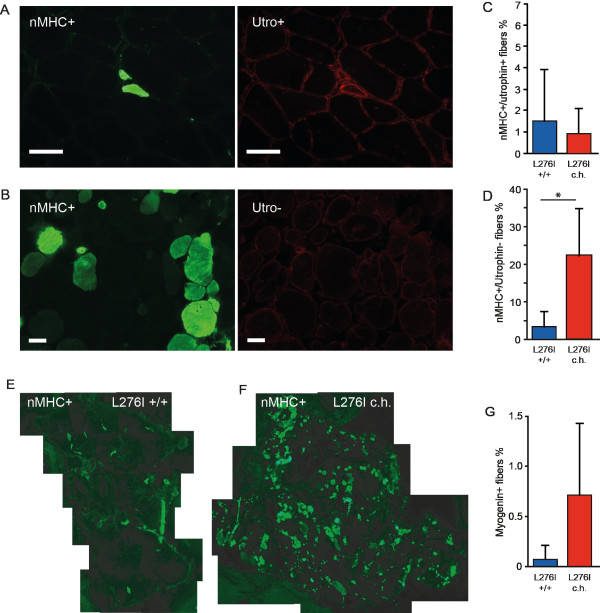
**Muscle regeneration in patients with LGMD2I**. (A) An illustration of newly regenerating fibers positive for neonatal myosin heavy chain (nMHC, green) and utrophin (red) (upper row), (B) Repair of mature fiber positive for nMHC and negative for utrophin (lower row). (C) The percentage of fibers positive for nMHC and utrophin did not differ between patients who were homozygous (n = 12) and those who were compound heterozygous (n = 5) for L276I. (D) The number of fibers positive for nMHC and negative for utrophin, as seen in fibers undergoing repair, was higher in compound heterozygous than in homozygous patients (*P *< 0.03). (E, F) nMHC expression in merged pictures of sections from (E) representative homozygous and (F) compound heterozygous patients. (G) The number of myogenin-positive nuclei trended towards an increase in compound heterozygous versus homozygous patients. Bar = 50 μm.

To assess the level of recent regeneration (within the previous 1 to 4 weeks), we stained for nMHC and utrophin. We noted that only small, newly regenerating myofibers stained positive for both nMHC and utrophin, whereas mature fibers with internal nuclei stained positive for nMHC only (Figure 3A-B). The number of small, newly regenerating fibers positive for both nMHC and utrophin did not differ significantly between the groups (Figure 3C). By contrast, the patients who were compound heterozygous for L276I had approximately seven times more nMHC-positive/utrophin-negative myofibers than did homozygous patients (Figure 3D, *P *< 0.03). A significant difference in nMHC expression between homozygous and compound heterozygous patients was seen (Figure 3E, F). To confirm this finding, we stained for the transcription factors that initiate the myogenic program in satellite cells. Although there were no MyoD-positive nuclei (data not shown), we noted that the number of myogenin-positive nuclei in the compound heterozygous patients trended towards an increase (*P *< 0.12), consistent with the nMHC finding (Figure 3G). Neither marker was found in any of the healthy subjects.

**Figure 4:**
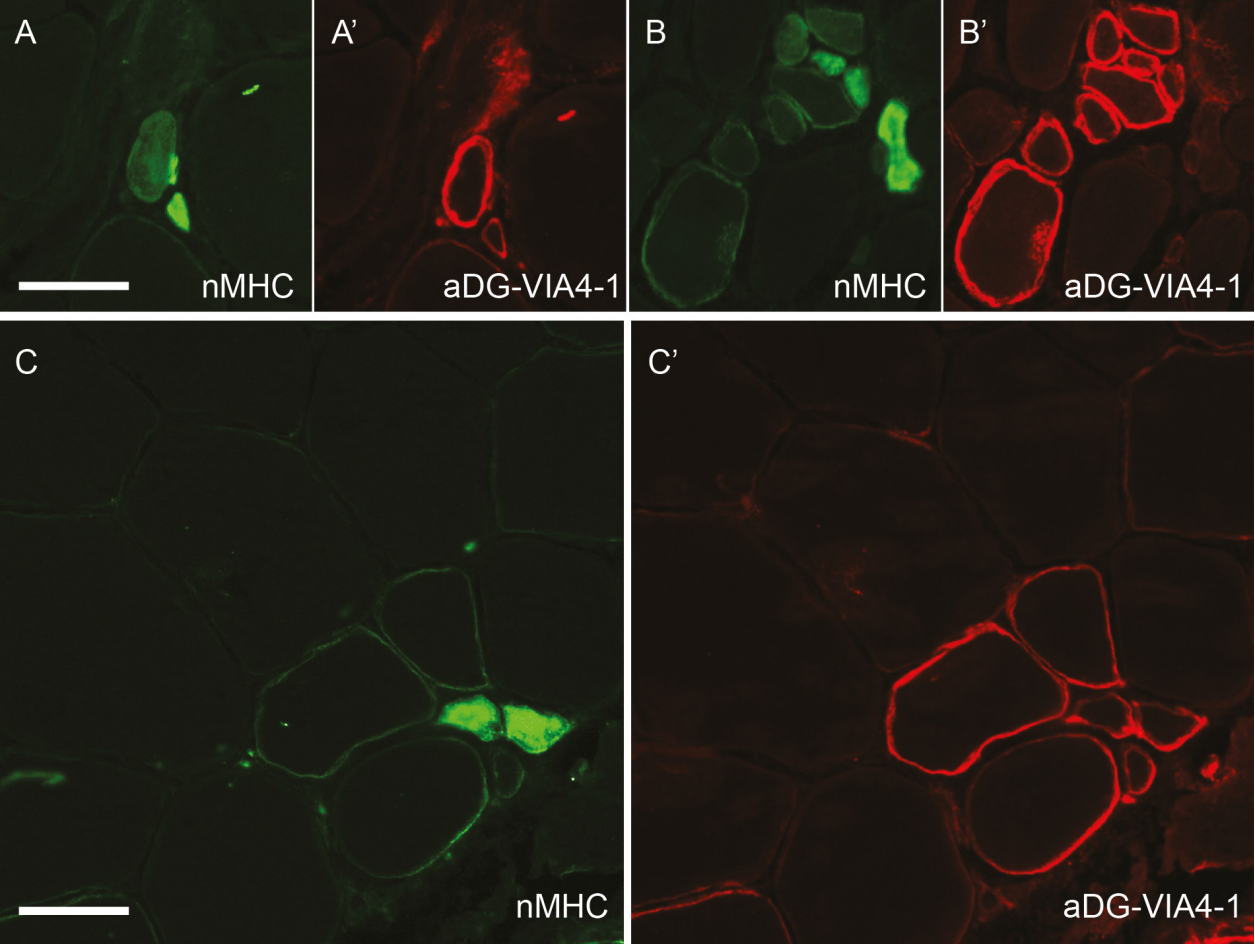
**Co-expression of neonatal myosin heavy chain (nMHC) and α-dystroglycan**. Many nMHC-positive fibers (A-C) also expressed α-dystroglycan (A'-C'). An inverse staining pattern was visible in small nMHC-positive fibers. (A) As the level of nMHC decreased, (A') α-dystroglycan increased. In a group of regenerating fibers, (B) the level of nMHC was low in all but two fibers whereas (B') all but one were α-dystroglycan positive. Within mature fibers, (C) the regenerating nMHC-positive fibers also stained positive for (C') α-dystroglycan. Bar = 50 μm.

Dual labeling with nMHC and glycosylated α-dystroglycan using the VIA4-1 antibody showed that the majority of small newly formed fibers staining positive for nMHC also stained positive for α-dystroglycan. There was an almost inverse staining pattern in groups of regenerating fibers (Figure 4A-C).

**Figure 5:**
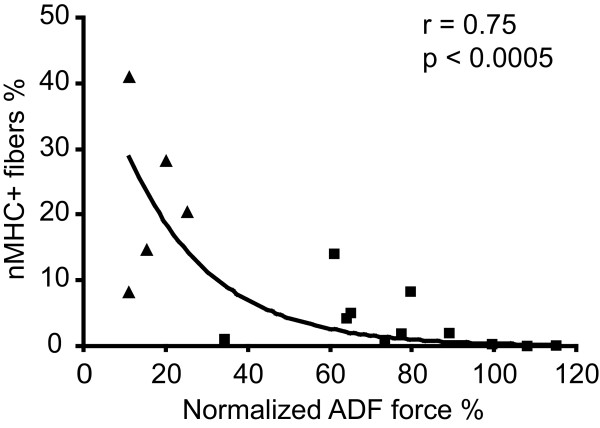
**Regeneration versus muscle strength**. There wass a significant relationship between regeneration in terms of number of neonatal myosin heavy chain (nMHC)-positive fibers as a function of ankle dorsiflexion (ADF) strength in patients with LGMD2I, reflecting greater need for muscle regeneration in patients who have lost muscle strength through muscle wasting (squares = patients homozygous for L276I; triangles = patients compound heterozygous for L276I).

We found no relationship between regeneration in terms of internally nucleated fibers and clinical severity and aging (data not shown). However, there was a significant relationship (*P *< 0.005 for all patients) between nMHC-positive fibers and clinical severity in terms of ankle dorsal flexion (Figure 5A) and duration of disease (*P *< 0.05, Figure 5B). In addition, we noticed a tendency for nMHC-positive fibers to decline with age in the L276I homozygous patients (*P *< 0.052, data not shown). Immunohistochemistry was carried out on biopsies from twelve homozygous and five compound heterozygous patients.

## Discussion

Most reports on LGMD2I have dealt with the clinical presentation and genetics of the disease, but little is known about the muscle morphology and regeneration of this disorder. In the absence of a substitute for FKRP, muscle fibers must rely on regeneration to counterbalance ongoing degeneration. In the present study, we determined the extent of this regeneration and whether the regenerative response is related to the clinical severity of LGMD2I, using some of the most commonly used markers for regeneration.

The key findings of the present study are that the level of early regeneration, as indicated by nMHC expression, was higher in patients who were compound heterozygous than in those homozygous for the L276I mutation, and that this regeneration was related to clinical severity. In addition, early regeneration tended to decrease with the duration of illness. Nevertheless, the apparent higher level of regeneration in compound heterozygous patients seems to be insufficient to maintain muscle mass, because the muscle wasting progresses more rapidly than in homozygous patients. The more severe phenotype in compound heterozygous patients was accompanied by a severe depletion of glycosylated α-dystroglycan. In these patients, α-dystroglycan staining was generally restricted to small regenerating fibers and large ring fibers, which is possibly caused by a failed regenerative process after contractures [18]. This expression is consistent with previous findings that dystroglycan is upregulated during regeneration [19]. In this study, the sometimes inverse staining pattern we saw with MHC and α-dystroglycan double-labeling suggests that as nMHC expression is reducing, the level of glycosylated α-dystroglycan is increasing. Small regenerating fibers are believed to have added membrane stability because of increased utrophin and α-7 integrin, hence the glycosylated α-dystroglycan may persist for a longer period in these fibers compared with mature fibers [20,21]. Ultimately, as the protective higher level of proteins important for regeneration starts to decline, the glycosylated α-dystroglycan presumably disappears. In Duchenne muscular dystrophy (DMD), utrophin, an autolog of dystrophin, can to some extent substitute for the missing dystrophin [22,23]. It is not known whether a functional substitute for FKRP that could delay the progression of LGMD2I exists, or whether all glycosylated α-dystroglycan originates from what basic functionality the mutated FKRP retains.

Although the L276I mutation affects FKRP, it retains some basic functionality, as suggested by the α-dystroglycan staining in homozygous patients. Carriers of single L276I mutations are asymptomatic, and patients homozygous for the L276I mutation often have a milder clinical course than most other patients with LGMD2I [24]. However, in patients who are compound heterozygous for the L276I mutation, the level of glycosylated, and thus functional, α-dystroglycan is further reduced. The glycosylations are vital for interaction with the ECM protein laminin α2 (merosin) thus maintaining sarcolemmal stability [25]. All the patients who were compound heterozygous for the L276I mutation in our study are wheelchair users, and are severely affected by the disorder. Patients who are compound heterozygous for L276I/P462S and L276I/Y307N have previously been described as having a more severe clinical presentation than patients homozygous for L276I [26,27], suggesting that, in most patients who are compound heterozygous for the L276I mutation, the other allele carries a mutation that renders FKRP even more dysfunctional than the homozygous L276I mutation itself.

Internally nucleated fibers are generally regarded as a marker for regenerating fibers after injury and muscular dystrophies, although there are rare exceptions to this (for example, centronuclear muscular dystrophy caused by impaired cohesion of the centrosome) [28,29]. In our study, we found that the number of internally nucleated fibers alone yielded very little information about the rate of regeneration, which probably relates to the fact that internally nucleated fibers can remain located in the cytoplasm, thus obscuring any difference in the rate of regeneration. This may explain why we did not find any significant relationship between internal nuclei and clinical severity, as expressed by the force in the muscle that we examined histologically. The absence of any trend towards change in internal nuclei throughout the age span in patients with the L276I mutation could be due to several reasons, including differences in physical activity, fairly constant degeneration during the progression of the disorder, too few patients over a large age span, or the level of regeneration not being limited by available satellite cells. This is in contrast to what others have found in healthy humans; however, those findings were based on an age-dependent decline in number of satellite cells and not on actual regeneration [30]. Immunostaining for nMHC is useful in assessing ongoing regeneration in dystrophic muscle [31-33]. This MHC isoform has a peak expression within 10 days in regenerating fibers, thus providing a more precise temporal picture of the ongoing regeneration [34]. Although nMHC has been shown previously to be present in atrophic fibers (for example, in spinal muscular atrophy due to denervation related lack of muscle maturation [35]), we did not observe any upregulation of nMHC in atrophic fibers. Other markers for recent regeneration exist, but no other study has identified a better marker than nMHC for recent regeneration [36]. By adding utrophin staining, which is present in varying degrees on regenerating fibers, but is strongly upregulated in newly formed fibers, it is possible to see what the preferred method of regeneration is: repair of fibers and/or production of new fibers [20]. An increase in nMHC-positive fibers suggests that regeneration is progressing at a significantly higher pace in the L276I compound heterozygous patients, and that regeneration in these patients is mainly repair of degenerating fibers (nMHC+/utrophin-) rather than development of new myofibers (nMHC+/utrophin+). We suggest that the increased level of nMHC reflects an increased rate of degeneration in severely affected patients, whereas patients with a milder phenotype apparently have little need for a high level of regeneration, because the resistance to structural stress is higher in the milder than in more severe form of LGMD2I, in which the fibers are more prone to degenerate. However, there is a possibility that delayed maturation is involved in the increased nMHC expression; that is, that the nMHC persists for longer than normal [37].

MyoD was not found in any of the muscles in this study. Presumably because of its short-lived action (<48 hrs) it is difficult to detect MyoD by immunostaining of muscle tissue [38]. By contrast, myogenin is expressed up to a week after differentiation of the satellite cells is initiated [39]. The fact that we found myogenin-positive nuclei in the tissue of compound heterozygous patients is consistent with the increased number of nMHC+/utrophin-myofibers, and can be interpreted as a constant need for myofiber repair, that is, a higher rate of regeneration. However, whether this ultimately leads to exhaustion of the satellite-cell pool in LGMD2I and contributes to the worsening of the disease remains to be shown.

The trend towards a decrease in nMHC-positive fibers with age in L276I homozygous patients may reflect an age-dependent rather than disorder-dependent decline in rate of regeneration. This seems inconsistent with the fact that we were unable to find a relationship between internal nuclei and age. However, activation of the satellite cells, the subsequent differentiation of the myoblasts leading to internal nuclei and the actual repair, although linked, are separate events, with the latter heavily dependent on protein synthesis (nMHC and other proteins), and age may affect these events differently. In patients who were compound heterozygous for the L276I mutation, the level of regeneration declined not only with age, but also with duration of illness. This implies that muscle repair decreases with time after onset, whereas muscle degeneration continues unabated, leading to progressive muscle wasting.

Recent advances in research of DMD therapy and research on sarcopenia have identified new opportunities for treating a wider field of muscular dystrophies by boosting the regenerative response, that is, by stimulating satellite-cell activation [40]. Knowledge of the existing regenerative response in LGMD2I may prove valuable, as differentiated regeneration may require different approaches for boosting the regenerative response to avoid adverse side effects, such as depleting the satellite-cell pool.

## Conclusions

In this study, we found that although patients with LGMD2I who were homozygous for the L276I mutation appeared to have the same level of regeneration as patients who were compound heterozygous for this mutation, as determined by level of internally nucleated fibers, the level of early regeneration, as judged by the level of neonatal myosin heavy chain, was significantly higher in the compound heterozygous patients. These results yield a more accurate picture of the ongoing degeneration-regeneration cycle.

## Abbreviations

α-DG: α-dystroglycan; FCS: fetal calf serum; FKRP: fukutin-related protein; LGMD2I: limb-girdle muscular dystrophy type 2I; L276I +/+: homozygous for the L276I mutation, L276I c.h.: compound heterozygous for the FKRP L276I mutation; nMHC: neonatal myosin heavy chain; PBS: phosphate-buffered saline.

## Competing interests

The authors declare that they have no competing interests.

## Authors' contributions

TOK conceived, designed the study and carried out the histology/immunohistology procedures as well data analysis and drafted the manuscript. SH carried out immunohistology and counted fibers, and performed the subsequent data analysis. MLS carried out force measurements on the patients. MS determined the mutations of the patients. JV provided biopsy material and helped to draft the manuscript. All authors read and approved the final manuscript.
